# A Mobile Self-control Training App to Improve Self-control and Physical Activity in People With Severe Mental Illness: Protocol for 2 Single-Case Experimental Designs

**DOI:** 10.2196/37727

**Published:** 2023-05-05

**Authors:** Tessa Dekkers, Tahnee Heirbaut, Stephanie E Schouten, Saskia M Kelders, Nienke Beerlage-de Jong, Geke D S Ludden, Jeroen Deenik, Yvonne H A Bouman, Hanneke Kip

**Affiliations:** 1 Centre for eHealth and Wellbeing Research Department of Technology, Human and Institutional Behaviour University of Twente Enschede Netherlands; 2 Optentia Research Unit North-West University Vanderbijlpark South Africa; 3 Technical Medical Centre Section of Health Technology and Services Research University of Twente Enschede Netherlands; 4 Department of Design Production & Management Faculty of Engineering Technology University of Twente Enschede Netherlands; 5 GGz Centraal Amersfoort Netherlands; 6 School for Mental Health and Neuroscience Maastricht University Maastricht Netherlands; 7 Department of Research Stichting Transfore Deventer Netherlands

**Keywords:** mobile health, mHealth, mobile apps, self-control, physical activity, accelerometry, single-case experimental design, mobile phone

## Abstract

**Background:**

Lack of physical activity is a common issue with detrimental consequences for the health of people with severe mental illness (SMI). Existing physical activity interventions show suboptimal effects as they require substantial cognitive skills, including goal setting and writing, whereas cognitive deficits are common in this population. To bolster the effectiveness of physical activity interventions, self-control training (SCT), in which users practice the ability to override unwanted thoughts and behaviors, can be used in addition. Recent research has demonstrated the initial effectiveness of a mobile SCT app, but this has not been studied in psychiatric clinical practice.

**Objective:**

This study aims to evaluate to what extent adding a mobile SCT app designed for and with people with SMI to a mobile lifestyle intervention aimed at increasing physical activity increases physical activity and self-control levels.

**Methods:**

A mixed methods approach incorporating 2 single-case experimental designs (SCEDs) and qualitative interviews was used to evaluate and optimize SCT. Overall, 12 participants with SMI will be recruited from 2 organizations offering outpatient and inpatient care to people with SMI. Each experiment will include 6 patients. SCED I is a concurrent multiple-baseline design across participants that explores initial effectiveness and optimal intervention duration. Using accelerometry and experience sampling questionnaires, participants’ physical activity and self-control will be monitored for ≥5 days from baseline, followed by the sequential introduction of Google Fit, the physical activity intervention, for 7 days and the addition of SCIPP: Self-Control Intervention App for 28 days. SCED II is an introduction/withdrawal design in which optimized SCT will be introduced and withdrawn to validate the findings from SCED I. In both experiments, the daily average of total activity counts per hour and the state level of self-control will serve as the primary and secondary outcome measures. Data will be analyzed using visual analysis and piecewise linear regression models.

**Results:**

The study was designated as not subject to the Dutch Medical Research Involving Human Subjects Act by the Medical Research Ethical Committee Oost-Nederland and approved by the Ethics Committee/domain Humanities and Social Sciences of the Faculty of Behavioural, Management, and Social Sciences at the University of Twente. Participant recruitment started in January 2022, and we expect to publish the results in early 2023.

**Conclusions:**

The mobile SCT app is expected to be feasible and effective. It is self-paced and scalable and can increase patient motivation, making it a suitable intervention for people with SMI. SCED is a relatively novel yet promising method for gaining insights into whether and how mobile apps work that can handle heterogeneous samples and makes it possible to involve a diverse population with SMI without having to include a large number of participants.

**International Registered Report Identifier (IRRID):**

PRR1-10.2196/37727

## Introduction

### Background

Approximately 1% of the world’s population lives with severe mental illnesses (SMIs) such as schizophrenia (20 million) or bipolar disorder (46 million) [1]. Although their experiences are diverse, many are faced with multifaceted psychosocial problems in daily life, including difficulties in employment, housing, relationships, and finances and physical health inequalities [2]. An especially poignant problem in this group is the lack of physical activity and increasing sedentary behavior. Compared with healthy controls, people with SMI are more sedentary, less physically active, and less likely to meet physical activity guidelines [3]. This has a broad range of detrimental consequences for the physical and mental health of people with SMI, including reduced quality of life, increased risk of cardiovascular disease, and increased symptom severity [2,4,5]. Fortunately, even minor improvements in physical activity can prevent problems with physical health and deterioration of psychiatric symptoms in people with SMI and, thus, improve their quality of life [2,6,7].

### The Role of Self-control in Successful Behavior Change

Clearly, there is an urgent need to improve physical activity in this extremely vulnerable group. However, existing physical activity interventions have several limitations when used in the population with SMI. First, many interventions are delivered by professionals, requiring additional time from already overworked staff [7,8]. For example, behavioral physical activity interventions targeted at people with SMI typically involve biweekly or weekly 60- to 120-minute sessions of counseling [9]. In addition, most physical activity interventions developed for people with SMI are not based on theory [9]. When they are, interventions are often solely underpinned by cognitive and reflective models of behavior change, including the Theory of Planned Behavior, Social Cognitive Theory, and the transtheoretical model [9-11]. Such interventions require substantial cognitive skills, including goal setting, reflecting on behavior, and writing, to produce effects [10]. This makes these interventions especially hard to use for people with SMI because of the vulnerabilities that are common in this target group related to cognitive deficits in memory and attention, low literacy rates, and apathy [12-17]. Finally, although behavioral theories are helpful in predicting behavioral intentions, these intentions do not necessarily relate to accompanying behavior changes, for example, increased physical activity [10,18]. This might explain why there is currently no reliable evidence that physical activity interventions result in better long-term physical health in people with SMI [9]. To address all the aforementioned issues, we need to better target physical activity interventions to vulnerable groups, including people with SMI.

This requires interventions that patients can work on independently; that do not require a high level of cognitive skills; and that target the intention-behavior gap that is common in interventions that are based on cognitive, reflective models. A promising focal point of these interventions is self-control—the ability to prevent or override unwanted thoughts or behaviors in favor of higher-order goals [19]. Studies have shown that self-control plays an important role in a broad range of goal-directed behaviors, including academic achievement, aggression regulation, and healthy eating and physical activity [19]. According to Friese et al [20], the role of self-control can be explained through three interacting components: (1) reflective processes such as personal goals, (2) impulsive processes such as automatic approach and avoidance tendencies, and (3) self-control abilities that allow for the control of impulsive processes and transfer of reflective processes into behavior. Owing to the large body of evidence that shows the relationship between self-control and health behaviors, self-control is an important element of many existing interventions. Even so, these mostly focus on self-control as a reflective process and teach reflective strategies such as goal setting, mental contrasting, and mental transformations [21], whereas interventions that address impulsive processes are underrepresented.

A conceptually different intervention is self-control training (SCT). SCT focuses on improving self-control abilities through repeated practice of self-control. In line with the impulsive processes described by Friese et al [20], individuals practice self-control by suppressing automatic tendencies in low-stakes areas for a predetermined period, often 2 weeks. A common paradigm is the use of the nondominant hand for everyday tasks such as writing or opening doors. This practice is then expected to transfer to behaviors for which self-control matters, such as physical activity. SCT is based on the strength model of self-control [22]. It states that all acts of self-regulation draw from a common, limited resource of self-control. Upon repeated exertion, this common resource becomes depleted, resulting in impaired inhibition and goal-directed behavior until the resource is renewed. Although this theory suggests that exerting self-control has negative short-term effects, it also states that the repeated use of self-control strengthens the capacity for self-control in the long run, akin to a muscle.

Traditionally, participants receive face-to-face or written instructions from researchers or health care staff to perform self-control tasks [23,24]. These paper-based versions of SCT consistently show that this practice results in increased self-control [24-26]. As such, SCT has multiple advantages in clinical practice. It is easy to administer, does not require much time and effort from participants, and can be a valuable addition to existing treatment and interventions by means of its focus on the automatic aspect of self-control. However, face-to-face delivery of SCT can take up the precious time of (health care) professionals. To prevent this, delivery of SCT can also be done digitally using either web-based applications or mobile apps.

A mobile app specifically has advantages for the delivery of SCT in clinical practice. First, an app is scalable and easy to implement as it can be accessed by many people without requiring time from already overworked health care staff [27]. Second, design principles such as gamification can be applied to support people who are not that motivated to use SCT. This is especially relevant in populations with low treatment motivation, such as psychiatric patients [28,29]. To involve these target groups, persuasive strategies can be embedded in the design of the app to increase adherence and engagement [30,31]. Adding persuasive features such as rewards, reminders, and personalization to an SCT app can support users in using the intervention as intended, resulting in more positive outcomes. As an app can increase motivation and does not require a high level of cognitive effort, it can be a suitable way to bolster self-control and, thereby, increase the level of physical activity in people with SMI. The first results of using such an SCT app demonstrated that, among 204 healthy young adults who used a mobile SCT app, 10 days of app-based SCT already significantly increased self-control, whereas participants in the control and email conditions showed no improvement [32].

Overall, a large body of research has shown that SCT strengthens self-control [24-26]. However, similar to the effect sizes of cognitive physical activity interventions [33], the effect sizes of SCT alone on behavior are small [25]. Hence, we argue that multiple complementary interventions should be offered that address reflective processes, impulsive processes, and self-control abilities to produce meaningful changes in health behavior. To illustrate, reflective interventions focused on goal setting can be used to create physical activity intentions and directly stimulate behavior change. SCT can be added to these interventions to address the impulsive processes related to self-control, thereby increasing individuals’ general self-control capacity and indirectly supporting behavior change. When general self-control capacity is high, individuals are more likely to inhibit the dominant response of remaining sedentary and are expected to instead go for a walk—in other words, they are more likely to put their intention of walking into practice. This suggests that SCT could be added to many existing cognitive interventions to increase participants’ self-control and, thus, decrease the intention-behavior gap and bolster the effectiveness of interventions. Surprisingly, despite the large body of evidence showing SCT’s potential [24-26,32], it has not been studied in combination with existing cognitive interventions yet. In addition, despite its straightforward nature, SCT has rarely been applied and studied in psychiatric clinical practice.

### Study Objectives

Therefore, this study further explores a mobile SCT app and the assumption that it can be used to bolster the effectiveness of existing physical activity interventions. It does so specifically in the vulnerable population of people with SMI. The primary objective is to assess the effect of adding a mobile SCT app to a mobile lifestyle intervention aimed at increasing physical activity (Google Fit) on physical activity levels in people with SMI. The secondary objective is to examine the effect of this combined intervention on self-control directly. Finally, we will explore how long SCT should be offered to produce beneficial outcomes on physical activity and whether and which individual characteristics of patients influence SCT effects.

## Methods

### Ethics Approval

This study has been designated as not subject to the Dutch Medical Research Involving Human Subjects Act by the Medical Research Ethical Committee Oost-Nederland (approval 2021-13232). Consequently, it has been approved by the Ethics Committee/domain Humanities and Social Sciences of the Faculty of Behavioural, Management, and Social Sciences at the University of Twente (approval 211301) and the research committees of Dimence Groep and GGz Centraal. In addition, this study has been registered at *CCMO Toetsingonline* from the Dutch Central Committee on Research Involving Human Subjects (file NL79450.091.21). This portal will also be used to report (serious) adverse events if they occur.

### Study Design

This study will be conducted using a series of 2 single-case experimental designs (SCEDs): a concurrent multiple-baseline design across participants (SCED I) [34,35] and an introduction/withdrawal design (SCED II). SCED is a family of high-quality experimental designs that can be used to draw causal inferences about the relationship between the intervention and the outcome [36,37]. In an SCED, the intervention is systematically manipulated across multiple phases of introduction and withdrawal while the outcome variable is measured repeatedly and frequently [36-38]. In this way, individual patients function as their own controls. An overview of both experiments according to the SCRIBE (Single-Case Reporting Guideline in Behavioral Interventions) guidelines [39] is provided in Table 1.

**Table 1:** Single-Case Reporting Guideline in Behavioral Interventions design elements of single-case experimental designs (SCEDs) I and II.

	SCED I	SCED II
Design	Multiple baselines across participants	Introduction-withdrawal
Duration	52 days	Approximately 42 days (28-70 days)
Sequence of phases	A-B-C+follow-up	B-C-B-C
Description of phases	Phase A—baseline: 5 days minimum; no intervention, only monitoring of physical activity and self-control Phase B—Google Fit: 7 days minimum; only Google Fit intervention is administered Phase C—Google Fit+SCT^a^: 28 days minimum; Google Fit and SCT intervention are administered simultaneously Follow-up: 3 days minimum; no intervention, only monitoring of physical activity and self-control	Phase B—Google Fit: 7 days; only Google Fit intervention is administered Phase C—Google Fit+SCT: 7-28 days minimum; optimal phase length is determined from findings of SCED I; Google Fit and SCT intervention are administered simultaneously
Main outcomes	Average TACs^b^ per hour, measured continuously throughout the trial (at least 5 measures per phase) Daily self-control, measured twice daily throughout the trial (at least 5 measures per phase)	Average TACs per hour, measured continuously throughout the trial (at least 5 measures per phase) Daily self-control, measured twice daily throughout the trial (at least 5 measures per phase)
Randomization	Baseline duration is randomized with restriction to have at least 5 baseline observation days and 3 measurements	Not randomized

^a^SCT: self-control training.

^b^TAC: total activity count.

There are several reasons for using SCED to evaluate interventions in people with SMI. First, SCEDs require only a small number of participants—typically 3 to 6. This makes it more feasible to recruit a sufficient number of participants and requires less time and financial resources from both staff and patients. Furthermore, SCEDs can handle heterogeneous samples as participants function as their own controls. This makes it possible to involve the diverse population with SMI, who may present with comorbidities and a broad range of psychosocial problems. Finally, SCEDs enable research into the working mechanisms of SCT, which is required [25] but nearly impossible to achieve through a randomized controlled trial (RCT) as an intervention is treated as a coherent whole, making it hard to open the black box of the studied intervention [40]. Overall, this makes SCEDs very suitable for the evaluation of interventions [41,42] and an excellent and feasible alternative to classic experimental designs such as the RCT.

In total, 2 SCED experiments are used to address different aspects of the overall research aim. SCED I is used to (1) determine the initial effectiveness of the intervention, (2) explore for which patients SCT may be most effective, and (3) determine how long SCT should be offered for optimal effects. The design of SCED I is A-B-C+follow-up, where A refers to the baseline phase, B is the physical activity intervention alone (Google Fit), and C is the combination of physical activity intervention+SCT intervention, followed by a follow-up in which patients are monitored but no interventions are administered. In addition, SCED I uses a mixed methods design incorporating qualitative interviews to provide insights into patients’ experiences with both the mobile SCT app and the participation in an extensive longitudinal study. To explore the onset and duration of SCT effects on self-control, SCT is offered for 4 weeks instead of the customary 2 weeks [43]. This information will be used to determine whether SCT should be offered 1, 2, 3, or 4 weeks in SCED II.

The main aim of SCED II is to validate the effectiveness of the optimized intervention—in terms of duration and experience—based on the findings from SCED I. The design of SCED II is B-C-B-C, where, again, B refers to the physical activity intervention alone and C refers to the combination of physical activity intervention+SCT intervention. In both experiments, 6 participants will be monitored for at least one baseline phase and one intervention phase, with each phase lasting at least 5 data points following SCRIBE and What Works Clearinghouse guidelines [36,37,44]. Given that physical activity is measured continuously and self-control is measured twice a day, this will result in at least 28 data points during intervention phase C in SCED I and between 7 and 28 data points during intervention phase C in SCED II.

### Randomization and Blinding

We will randomly assign participants to different baseline, intervention phase B, and follow-up durations in SCED I. All are drawn a priori using the web-based random number generator Research Randomizer [45] with the restriction of having at least 5, 7, and 28 baseline observation days, respectively. In SCED II, phase length will not be randomized as the optimal intervention length will be derived from SCED I. Phase order will not be randomized in both experiments as we are interested in the additive effect of SCT over Google Fit only.

Owing to the nature of the intervention, in which apps are used by participants, it is not possible to blind participants to the phase. The researchers responsible for supporting participants in transferring between phases will not be blinded to the phase for the same reason. Where possible, researchers only involved in data entry, processing, and analysis will be blinded to the phase to improve the reliability of the findings [36].

### Participants

A total of 12 people with SMI will be recruited from the clinics of 2 large Dutch mental health care organizations: Dimence Groep and GGz Centraal. To meet quality standards, an SCED should include at least 3 participants [37,44,46]. To account for the high dropout rates common in the psychiatric population, we will include 6 participants in both SCEDs. Participants will receive €50.00 (US $56.00) for taking part. To promote continued commitment to the longitudinal design, participants will receive €10.00 (US $11.00) at the start, €20.00 (US $22.50) midway, and €20.00 (US $22.50) at the end of the study.

Participants need to meet the inclusion criterion of having an SMI, including but not limited to schizophrenia, bipolar disorder, and major depression, following the Dutch consensus definition of SMI [47]: (1) requires treatment (ie, no symptomatic remission), (2) is associated with severe limitations in social or societal functioning (ie, no functional remission) that result in and are caused by a psychiatric disorder, (3) is not transient (ie, is structural, chronic, or long-term [at least several years]), and (4) requires coordinated care from professional caregivers in care networks to realize the treatment plan.

As the study will be introduced only to people currently receiving care for their SMI at an outpatient clinic or residing in an inpatient clinic, all participants will, on principle, be expected to meet the consensus definition, and no specific diagnosis will be determined. If doubt exists about whether a participant meets this criterion, the health care provider responsible for the patient (Dutch: *persoonlijk begeleider*) will determine whether the participant has an SMI following the consensus definition. In addition, participants must be aged ≥18 years, receive outpatient treatment or reside in an inpatient clinic for at least the data collection period, have sufficient opportunity to be physically active (eg, not be confined to a bed or bedroom), have an intention to improve physical activity as assessed using the single-item assessment of stages of change adapted for healthy behavior [48,49], and provide voluntary consent to participate. Participants are excluded if they do not want to (further) improve their physical activity. This is due to the nature of the SCT intervention—SCT supports putting an existing goal into practice (ie, tackling the intention-behavior gap) but does not support patients who are unwilling to improve physical activity. In a similar vein, SCT is also not expected to be effective for people who already meet their physical activity goals. Participants are also excluded if repetitive behavior may potentially aggravate a patient’s disorder or symptoms (eg, obsessive-compulsive disorder) as evaluated by the health care provider or patient. Owning a mobile phone is not an inclusion criterion as participants who do not own a mobile phone will be provided one for the duration of the study.

To recruit participants, the study will be introduced to patients receiving treatment or residing in the clinics via short presentations and flyers distributed by the researchers. We specifically decided to do the initial introduction to the study via researchers rather than the health care provider to avoid any (perceived) role conflicts between health care providers and patients, for example, when the health care provider also makes decisions about patients’ leave in a forensic psychiatric context. If patients are interested in the study, they will be able to sign up directly with the researcher (in person or by email), after which we will discuss with their health care provider whether participation is safe, appropriate, and feasible for this patient.

When possible, only 1 patient from each clinic will be selected. This ensures that SCT is tested in different types of patients, staff, and clinics and prevents social comparison effects as patients from the same clinic could challenge each other to be more physically active. Patients not selected will be invited to participate in SCED II or, if not possible, will be offered to use the intervention after the study.

### Interventions

The interventions consist of 2 mobile apps designed to improve physical activity. The first is SCIPP: the Self-Control Intervention App, which was specifically designed for this study, and the second is Google Fit, a mobile lifestyle intervention aimed at increasing physical activity.

#### SCIPP: the Self-Control Intervention App

SCIPP is a mobile app based on the pen-and-paper format of SCT [24-26]. SCIPP is available on Android and provides users with a new daily challenge for 14 days. This daily challenge asks users to perform an everyday activity—such as opening the door or turning on the light—with their nondominant hand. After accepting the challenge, users are reminded of the daily challenge 4 times a day. After 14 days, the app notifies the user that the training has concluded. By restarting the app, the intervention can be used for a longer period—in the case of this study, up to 28 days.

**Figure 1:**
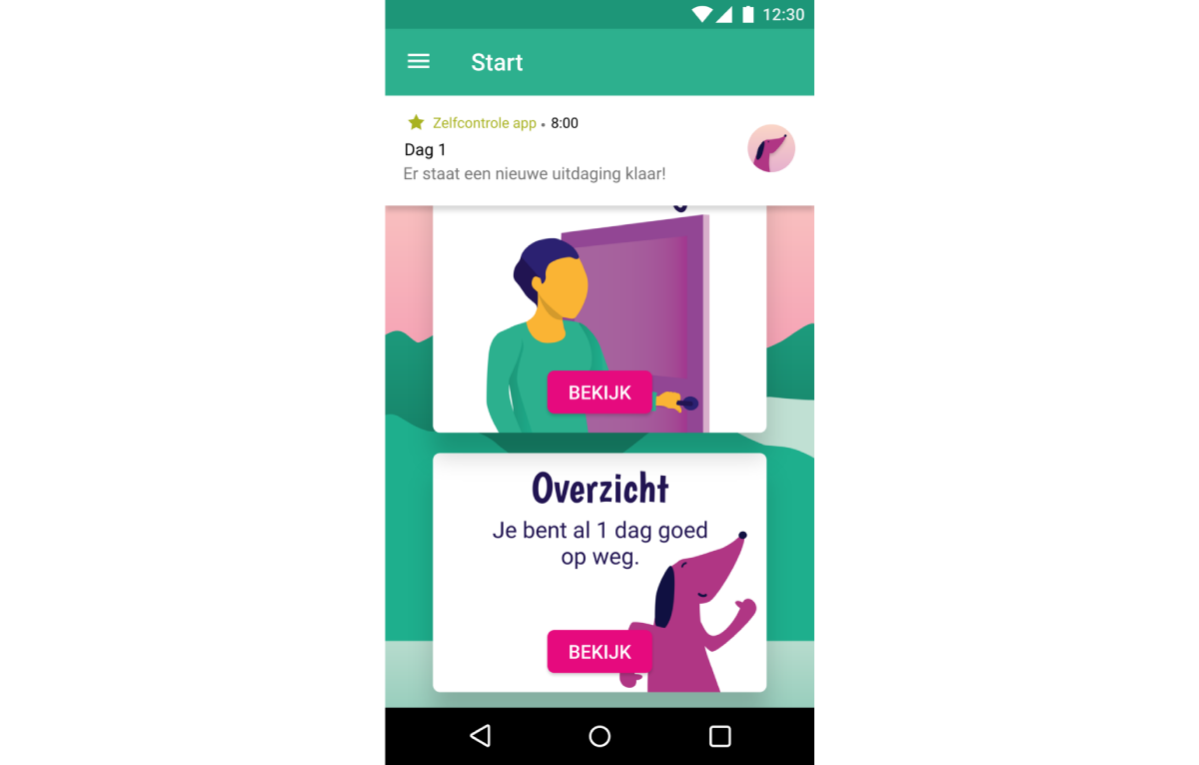
Screenshot of daily challenges with reminders in SCIPP: the Self-Control Intervention App. Daily challenges are easily found on the main page and explained visually. Users receive 4 daily reminders of the daily challenge.

**Figure 2:**
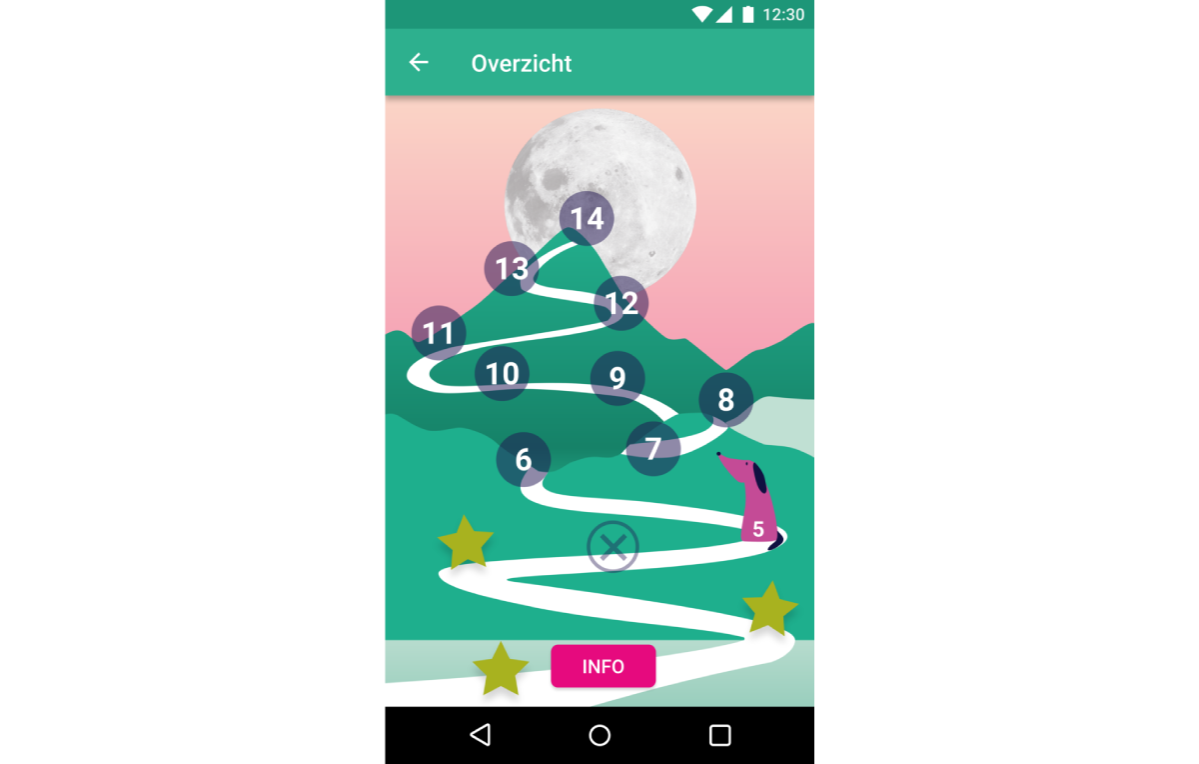
Screenshot of the process page with a winding mountain path in SCIPP: the Self-Control Intervention App. Users self-monitor daily challenges. As behavior change is an unpredictable process, the mountain visualization includes ups and downs, and participants can also report when they were not able to do a challenge.

**Figure 3:**
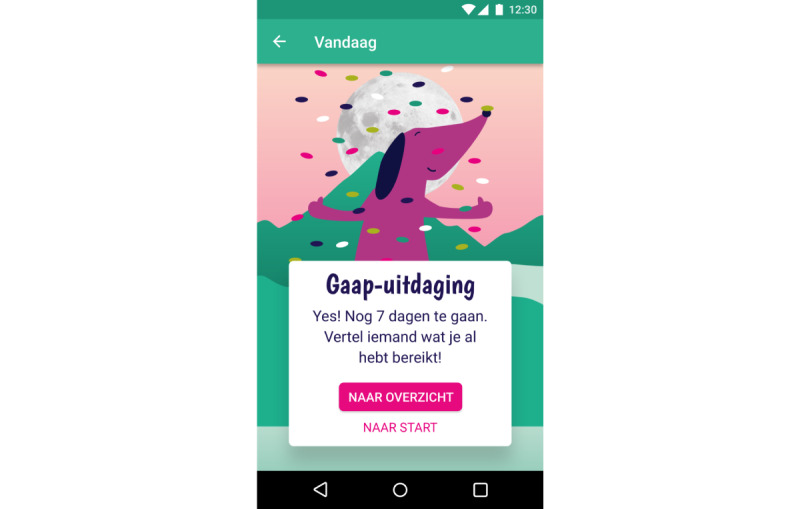
Screenshot of virtual coach Scipp in SCIPP: the Self-Control Intervention App. Virtual coach Scipp praises users for completing challenges. She also frames behavior change as a shared process and invites users to celebrate success with their broader social network after certain milestones, such as the first successful day of self-control training.

**Figure 4:**
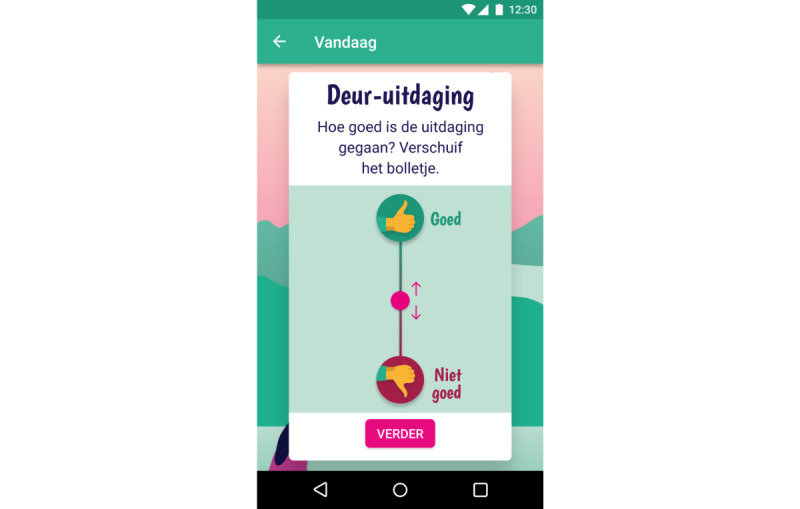
Screenshot of providing feedback on challenge difficulty in SCIPP: the Self-Control Intervention App. Users can set their own motivation for training self-control and provide feedback on the difficulty of the challenges. Once training is completed, this initial goal is shown to the users and can be adapted if needed.

We have previously established the feasibility and preliminary effectiveness of mobile SCT in young adults [32]. This study showed that self-control did improve in participants who used an SCT app, whereas self-control did not increase in participants who received SCT via email and participants in a control group that did not receive SCT. The app was then redesigned with and for people with SMI to meet their cognitive capabilities and preferences following a participatory design study [50]. It meets the following requirements for suitable SCT from the patients’ perspective: (1) behavior change is framed as a shared, unpredictable process; (2) visual rewards and explanations are given; (3) the app uses reduced textual and numerical information; and (4) the app provides accessible means for feedback and personal goals. In addition, the app sends reminders throughout the day to remind users of the daily challenge. This was considered specifically important as reminders appeared to be the main advantage of an app over email in the previous study [32]. Participants in the app condition of the previous study appreciated the reminders and even requested more, whereas participants in the email condition indicated that they often forgot about tasks and would have liked to have reminders. Finally, the SCT app also incorporates several other persuasive features to promote engagement and adherence [30,51,52], including reduction, self-monitoring, social role, praise, personalization, real-world feel, and liking. Reduction and self-monitoring features reduce complex behavior in simpler, smaller tasks and allow users to track their performance on these tasks. In the SCT app, this means that users monitor daily self-control challenges (Figures 1-2). Social role and praise enable users to form a social relationship with the app, which provides praise when the target behavior is performed. In the SCT app, this is embedded in the virtual coach Scipp (Figure 3). Personalization and real-world feel were incorporated through tangible, personalized goals and feedback (Figure 4), whereas liking refers to the look and feel of the app and is incorporated into the overall minimalist, colorful esthetic used.

#### Google Fit

Google Fit is a freely available mobile health tracking platform developed by Google in collaboration with the World Health Organization and the American Heart Association. Google Fit can be considered a goal-directed, cognitive intervention that incorporates various behavior change techniques to motivate the user to be more active. These include the ability to set a personal activity and step count goal, self-monitor activities in a personal dairy, and receive customized feedback based on health and activity history. To monitor physical fitness activities, Google Fit uses sensors from the user’s mobile device or external activity trackers. It translates each minute of moderately intense activity into a Heart Point, and more intense activities result in more points. In addition, the number of steps is tracked. It is available on the Android and iOS operating systems, but in this study, only the Android version will be used.

Google Fit was selected as the physical activity intervention in this study in line with the rationale provided in the Introduction section—it does not require additional support from staff; it is based on reflective processes of self-control (ie, a focus on cognitive processes such as goal setting) and, thus, can be complemented with the novel SCT intervention; and it is likely suitable for the cognitive abilities of people with SMI. The latter was determined based on the assessment of the Regional Public Health Services of the Netherlands, which assessed Google Fit as reliable, theory-informed, secure, and user-friendly, including short, concrete text free of difficult jargon or medical terms [53]. Although no evidence is available regarding the effectiveness of Google Fit in increasing the physical activity of people with SMI, it has been successfully used in people with poor mental health [54].

### Material and Measures

#### Overview

The primary study parameters are daily physical activity and self-control. Daily physical activity will be measured using a wearable accelerometer, the ActiGraph GTX+ [55,56]. Daily self-control will be measured via experience sampling.

#### Physical Activity

The ActiGraph GT3X+ and ActiLife software (version 6.8.0; ActiGraph) will be used to measure and analyze physical activity. The accelerometer is worn on the right hip and held in place with an elastic strap between 2 belt loops. Patients without belt loops will use a pouch pinned at the same location. The ActiGraph GT3X+ has a high inter- and intra-instrumental reliability [57] and validity [56]. It has been used successfully in previous studies with people with SMI to examine physical activity and sedentary behavior [6,58,59]. None of these studies reported usability, safety, or other issues related to the use of the ActiGraph GT3X+ in this population, which makes it a reliable, valid, and appropriate choice for measuring physical activity in this study.

Physical activity is operationalized as the daily average of total activity counts per hour, which is a continuous and detailed outcome variable of physical activity [6]. More counts indicate a higher level of physical activity. Following existing procedures for valid measurement of physical activity using the ActiGraph GT3X+ in this population, a wear time of >6 hours per day for at least 3 days will be used as the criterion for sufficient measurement [6,58].

#### Daily Self-control

To distribute questionnaires, the mobile survey distribution platform Ethica Data [60] will be used. Ethica is a web-based platform that is designed for researchers to create, modify, and distribute momentary surveys for experience sampling research. It allows researchers to obtain and view the data of the participant in real time to identify possible errors while the study is still running. Furthermore, it amplifies the ecological validity, possibilities, and reliability of frequent data collection [61]. Participants use their smartphones to complete the surveys. This reduces strain on participants as they do not have to carry with them additional study-related materials. Finally, trigger logistics help reduce participant burden by sending automatic reminders to fill out surveys [62].

Daily self-control will be measured using 2 items from the State Self-Control Scale [63] and 2 items specifically developed for this study. Participants use a 5-point slider ranging from 1 (*not at all*) to 5 (*very much*) to reflect on recent self-control experiences. All questions start with “In the past couple of hours...” Participants answer questions about decision-making (“it’s hard to make up my mind about even simple things”), subjective feelings of ego depletion (“I had less mental and emotional energy than I normally have”), goal-directed behavior (“I was able to do what I had planned”), and inhibitory control (“I was able to resist temptations”). We have previously used this questionnaire to measure daily self-control in students [64] and rephrased the items for people with SMI to improve clarity and brevity. A small pilot study with 5 patients receiving outpatient psychotherapeutic care showed that the rephrased items had high face validity, feasibility, and comprehension [65].

#### Trait Self-control and Cognitive Control

Generalization measures assess the intervention effect on untrained tasks and are important to establish the external validity in SCEDs [36]. The SCRIBE guidelines [37] recommend that generalization measures are taken repeatedly, although not as frequently as the main outcome measures. In this study, trait self-control and cognitive control are used as generalization measures and assessed every 5 days (at least once per phase). This combination of measures is used as self-control is a multifaceted construct and it is recommended to use more than one type of measurement instrument to assess it [66].

Trait self-control is measured using the Brief Self-Control Scale (BSCS). The BSCS is a validated, brief, and psychometrically sound measure of individual differences in self-control that consists of 13 items [19,66,67]. The BSCS measures several aspects of trait self-control, including inhibitory and initiatory control [68]. Participants rate the extent to which a statement, for example, “I am good at resisting temptation,” matches them on a 5-point Likert scale ranging from 1 (*not at all*) to 5 (*very much*). A total score for self-control will be created by taking the sum of all items after recoding negatively phrased items (Cronbach α=.83-.85 [69]). Higher scores indicate higher levels of self-control, which has a medium effect on behavioral outcomes, showing criterion validity [70].

Cognitive control will be measured using the go/no-go task. This is a well-studied measure of cognitive control and has been used in previous research to assess self-control [32]. In the go/no-go task, participants are instructed to respond to target stimuli but must refrain from responding to nontarget stimuli, which requires suppressing a behavioral response. In this study, we will use average reaction time as the main parameter of cognitive control, with shorter reaction times reflecting more self-control [32,71].

#### Sociodemographics and Covariates

To describe the characteristics of the participants, we will collect information about their age and gender. To explore possible covariates or moderators of the intervention effect, we will collect data on body weight, height, stage of change, and clinic type (outpatient or inpatient) and monitor intervention use (number of completed challenges and self-reported success of each challenge).

#### Experience and Feasibility Measures

Once the study is complete, patients’ perspectives will be gathered during short semistructured interviews. The interviews will focus on participants’ experiences with the SCIPP app, possible points of improvement, and their perceived effectiveness of SCT. This information will be used to optimize digital SCT training as well as understand the feasibility of using SCED methodology in people with SMI.

### Procedure

Participants will be visited by a member of the research team 1 day before the start of the experiment. The researcher will help the patient install all necessary apps on the patient’s mobile phone. Next, the researcher will fit the accelerometer to the patients’ hip or belt and demonstrate how to take it off. Finally, the researcher will help participants fill out the baseline questionnaires.

During the baseline phases, physical activity and self-control will be monitored, but no interventions will be administered. To monitor physical activity, the patient will wear the accelerometer each day from 9 AM to 11:59 PM except when sleeping or during activities that involve water, such as showering or swimming. To measure daily self-control, they will be prompted to fill out a morning survey between 11 AM and 1 PM and an evening survey between 7 PM and 9 PM. Reminders for both surveys will be sent after 30 and 60 minutes.

At the start of intervention phase B, the researcher will visit the patient and help set up Google Fit by entering their activity goals (number of steps and number of Heart Points), wake-sleep schedule, weight, and height. Next, the researcher will demonstrate where to find and how to use the primary functions of Google Fit. This will enable patients to self-monitor their activity and receive personalized coaching. During the withdrawal phases of SCED II, the researcher will also deactivate the SCT app and explain to the patient that this app will not be used for the next phase.

At the start of intervention phase C, the researcher will visit the patient and help set up SCIPP by entering the personal goal and dominant hand. The researcher will also inquire about their experiences with participation so far and provide additional instructions regarding Google Fit, the accelerometer, or Ethica, if required. The next day, the patient will receive the first self-control challenge and continue to receive and complete challenges throughout the phase. The researcher will return every 7 days to answer questions and, if necessary, reset the app to extend use up to 28 days until the phase is completed.

Follow-up follows the same procedure as the baseline phases. The researcher will visit the patient to disable all apps on the patient’s phone except Ethica, which is used for data collection. Once the study is completed, the researcher will make a final visit to collect the accelerometer and reactivate any apps that the patient wishes to continue using. In SCED I, the researcher will also conduct the postintervention interview during this visit.

### Data Analysis

#### Overview

Data analysis will include both visual and supplementary statistical analyses of the time-series data for both SCED I and II [36,42]. Data analysis will be performed using Microsoft Excel (Microsoft Corp), the web-based Tau-U calculator accessible via Single Case Research [72], and R (version 1.4; R Foundation for Statistical Computing) [73] and separately for the primary and secondary outcomes.

To prepare the data for analysis, we will score the primary outcome of physical activity as the daily average of total activity counts per hour. The secondary outcome of daily self-control will be scored as the daily average of the 4 self-control items. Next, we will use the median average deviation to identify and remove outliers. The median average deviation uses the median to identify scores that deviate 3.5 times from the median as outliers using a 95% CI. It is specifically recommended for use in small-sample studies, including SCEDs [74].

As both experiments collect continuous data that are averaged on a daily level, our missing data approach primarily consists of making full use of data that are available. To illustrate, self-control will be measured in the morning and evening. If the morning data are missing, the afternoon data will be used to calculate the level of self-control for that day. If no data are available on a given day, we will use multiple imputation to impute missing data. Multiple imputation methods have recently been developed specifically for use in SCED studies and have several advantages over other missing data approaches, including retention of collected data, maintenance of the design structure of an SCED, reduction of bias, and the ability to capture and communicate uncertainty regarding imputed scores [75].

#### Visual Analysis

SCED data are primarily interpreted visually [36]. To do so in this study, physical activity and state self-control data will be presented as a time-series graph, with days on the horizontal axis, physical activity or self-control on the vertical axis, and phase changes presented as vertical lines. As recommended by the What Works Clearinghouse guidelines [44,46], we will consider 6 features to examine within- and between-phase data patterns visually: level, trend, variability, overlap, immediacy, and consistency. The phase contrasts to be compared in SCED I are A versus B (baseline with Google Fit only), B versus C (Google Fit only with Google Fit+SCT), and A versus C (baseline with Google Fit+SCT). The phases compared in SCED II are B versus C (Google Fit only with Google Fit+SCT).

Within phases, *level* refers to the mean of all data points in 1 phase and is used to compare changes between phases [36]. *Trend* is the slope of the best-fitting trend line for all data in a phase and is used to determine trends toward (spontaneous) improvement or decline. *Variability* expresses the SD of data about the trend line and is used to calculate the percentage of overlap between data collected in different phases.

Between phases, *overlap* is the proportion of overlap between data from 2 phases, where less overlap indicates stronger intervention effects. The specific nonoverlap index we will use is the Tau-U summary index [76], which is a family of 4 indexes that calculate nonoverlap in relation to the trend. It is expressed by “the percent of data that improve over time considering both phase nonoverlap and Phase B trend, after control of Phase A trend” [76]. Using Tau-U allows for the exploration of, for example, how much the physical activity of a patient has increased after using the SCIPP app while considering a possible increase in physical activity levels that may have already been initiated by Google Fit during the previous phase.

*Immediacy* compares the extent to which the level, trend, and variability of the last and first 3 data points of subsequent phases are discriminably different. In SCED I, immediacy is used to examine whether SCT effects are immediate or cumulative and to make decisions about the appropriate phase length in SCED II. Finally, *consistency* involves examining whether data patterns (level and trend) are consistent in phases with the same condition, where more consistent patterns provide greater certainty for a causal relationship between the intervention and outcome. This will be used in SCED II to examine whether physical activity reaches similar level and trend effects in both B phases (Google Fit only) and both C phases (Google Fit+SCT).

#### Piecewise Linear Regression Analysis

To supplement the visual analysis, we will conduct piecewise linear regression analyses (PLMs) using the *scan* package [77]. PLM is an approach to analyzing time-series data that are segmented into phases, including SCEDs [77,78]. The PLM model calculates 4 parameters (intercept, trend, level, and slope) separately for data from each phase, allowing for a comparison between phases. In this case, *intercept* refers to the performance at the start of the study, *trend* effects refer to the continuous increase in the behavior over time, *level* effects refer to the constant and instant effect of the intervention, and *slope* effects refer to the change in continuous increase initiated by the intervention.

Using the PLM, we will construct a linear model for each individual patient, including a *P* value, *R*^2^ effect size, and beta weights for trend, level, and slope effects. We will use a cutoff of *P*<.05 to determine significant effects.

In SCED I only, we will also construct a multilevel PLM to aggregate single-case findings across all participants included. Aggregation improves the external validity of the findings by providing overall trend, level, and slope effects and allows for the exploration and quantification of moderation effects using random slopes [77]. First, we will include random slopes in the multilevel PLM to examine whether trend, level, and slope effects differ significantly between participants. If this is the case, we will subsequently consider the interactions between body weight, height, stage of change, clinic type, intervention use, trait self-control, and cognitive control as potential moderators of the effect. As there are no existing hypotheses about moderation, we will not use confirmatory *P* values in this analysis but merely report any findings as preliminary and exploratory.

#### Reliable Change in Generalization Measures

We will explore individual changes in generalization measures of trait self-control and cognitive control by comparing the means of pre- and postintervention scores obtained during baseline, end of intervention phase C, and follow-up.

#### Qualitative Analysis of Experience

Interview data will be analyzed inductively using thematic analysis [79] to organize data into overarching themes related to usability and feasibility of the SCIPP app and SCED methodology according to people with SMI.

## Results

Funding for this study was obtained in July 2020 by The Netherlands Organisation for Health Research and Development (project 555003023). It was approved by the Ethics Committee/domain Humanities and Social Sciences of the Faculty of Behavioural, Management, and Social Sciences at the University of Twente in November 2021 and the research committees of the participating mental health care facilities in January 2022. Recruitment for the study started in January 2022, and 6 participants have been included as of March 2023. We expect to publish the results in summer 2023.

## Discussion

### Expected Findings

Earlier studies have shown that SCT using the nondominant hand paradigm can increase self-control in healthy populations [24-26]. Self-control is a key mechanism in translating physical activity intentions into practice [80,81]. Therefore, interventions targeting self-control may be a viable strategy for improving physical activity in people with SMI. Many people with SMI experience poor physical health yet are barely targeted in preventive and health promotion research, leading to large systemic inequality [82]. In addition, people with SMI face a number of social, cognitive, physical, psychological, and sensory impairments, which may make cognitively based physical activity interventions less accessible [83,84]. However, further studies are needed to evaluate the effect of combining SCT and regular physical activity interventions on physical activity in this target group. This study will provide initial insights into whether this is the case.

SCIPP, the SCT app that will be used in this study, is expected to be feasible, effective, and suitable for people with SMI. First, it brings SCT to the digital era as it is delivered via a mobile app. Among other things, an app can be used by patients individually, on their own time, and at their own pace; is scalable; and can increase patient motivation and adherence, making it a suitable instrument to target people with SMI [85]. Previous research has indeed shown that there is a need for well-designed mobile apps to further improve treatment of hard-to-involve patient populations [86]. Second, based on earlier research on SCT and a specific study on an SCT app, it is expected that SCIPP will be effective. We have previously developed and pilot-tested a functioning prototype of an SCT app aimed at students and found that self-control increased after just 10 days [32]. Even though this was studied in a different target group, it still shows the potential of this app to bolster self-control as it is expected that the underlying mechanisms of self-control practice are similar across populations. The findings of this previous study were used to further develop the SCT app, resulting in SCIPP. This study especially highlighted the importance of reminders to ensure that participants did not forget about the SCT tasks throughout their day. Second, SCIPP was specifically designed with and for this target group following a participatory design process in which patients were structurally involved in app development via 3 design workshops. Products designed through a participatory design approach often have a better user experience and usability as they fit more closely with users’ needs [84,87]. Indeed, usability tests with 3 patients with SMI who were not involved in the design workshops showed that patients had a high level of satisfaction and intention to use SCIPP. Interventions are expected to benefit from this improved fit in multiple ways, including increased effectiveness [87], adherence [88,89], and adoption. Therefore, we expect that the mobile SCT app will provide an effective and suitable way to train self-control in the population with SMI. This should not be considered a stand-alone psychological treatment, but following this trial, the app could be implemented alongside any intervention aimed at improving the lifestyle of people with SMI to bolster its effectiveness.

To examine SCIPP in concert with Google Fit, we will use 2 SCEDs. The classic RCT is—despite its shortcomings—still the most dominant design for eHealth evaluation [41]. However, assembling the homogeneous population that is required for controlled trials is difficult or even impossible to achieve because of the relatively high number of patients with comorbidities. The fact that people with SMI are considered difficult to involve in larger trials such as RCTs may explain the lack of studies on their physical activity levels. This illustrates a paradox: even though vulnerable populations require more research to improve care, they are very hard to involve in classic study designs, which results in an underrepresentation in scientific literature, which in turn further aggravates inequalities [90]. SCEDs provide a good method to address this paradox as they allow for high-quality experimental research by intensively monitoring a limited number of patients. The first studies show that SCEDs are very suitable for the evaluation of eHealth interventions [41,42] and offer an excellent and feasible alternative to more classic experimental designs such as the RCT. However, although SCEDs appear to be a fitting research design, they have not been used to study eHealth physical activity interventions developed for people with SMI. This implies the need for more studies that apply SCEDs. Consequently, this project will apply 2 SCEDs to evaluate the effectiveness of combining a cognitive-based physical activity intervention with a mobile app to train self-control. This study will also explore people with SMI’s experiences of being involved in such an extensive longitudinal study. As such, these findings will also allow other researchers to learn the benefits and pitfalls of applying this relatively novel design to the evaluation of other eHealth interventions.

### Strengths and Limitations

A major strength of this study is the combination of 2 approaches to SCED: the multiple-baseline design across participants and the introduction/withdrawal design. The first experiment will allow us to demonstrate whether mobile SCT is effective, for whom it may be most effective, and for how long it should be offered for optimal effects. In addition, the qualitative interviews provide insights into how to optimize the mobile SCT app as well as insights into participation in an extensive longitudinal study from the perspective of people with SMI. This knowledge will be directly applied to the second experiment, in which the optimized intervention will be offered to a new set of patients to validate these findings.

Second, the study also uses accelerometry to assess the physical activity of people with SMI objectively. The use of accelerometry data substantially improves the reliability, validity, and level of detail of data on sedentary behavior and physical activity compared with often-used self-reports in which physical activity levels are consistently overestimated [91-93].

The main limitation is that there is no certainty that people with SMI will persist in SCT, faithfully wear the accelerometer, and provide answers to the daily questionnaires for the considerable study duration of 7 weeks. Nonadherence is a major challenge for any longitudinal study, including N-of-1 designs such as SCED [94]. However, it is specifically challenging in people with SMI [95], and studies suggest that low self-control may also further contribute to low adherence [96]. As this study is both longitudinal and will target individuals with SMI (who commonly have lower self-control than the general population [97]), nonadherence may thus be expected. To address this limitation, we will take additional measurements to avoid complete loss of data (eg, 2 daily measures of self-control instead of 1), schedule regular phone calls and visits by the researcher, and offer financial incentives for continued participation in the study, all strategies associated with better adherence [98-100]. Explicitly reporting on the success of these strategies both from a quantitative (eg, use data) and qualitative (eg, patient interviews) perspective will also help other researchers make more informed decisions about the design of SCED studies specifically in the population with SMI.

## Abbreviations

BSCS: Brief Self-Control Scale
PLM: piecewise linear regression analysis
RCT: randomized controlled trial
SCED: single-case experimental design
SCIPP: Self-Control Intervention App
SCRIBE: Single-Case Reporting Guideline in Behavioral Interventions
SCT: self-control training
SMI: severe mental illness

## Data Availability

All data generated during the study will be coded and handled confidentially and anonymously in accordance with the European Union General Data Protection Regulation and the Dutch General Data Protection Regulation Implementation Act. The coded data will be stored at the University of Twente for a period of 15 years. During this period, coded data will be available through the corresponding author upon reasonable request. After the period of 15 years, data will be stored in long-term storage at the Data Archiving and Networked Services by the Royal Netherlands Academy of Arts and Sciences.

## References

[1] GBD 2017 Disease and Injury Incidence and Prevalence Collaborators (2018). Global, regional, and national incidence, prevalence, and years lived with disability for 354 diseases and injuries for 195 countries and territories, 1990-2017: a systematic analysis for the Global Burden of Disease Study 2017. Lancet.

[2] Firth J, Siddiqi N, Koyanagi A, Siskind D, Rosenbaum S, Galletly C, Allan S, Caneo C, Carney R, Carvalho AF, Chatterton ML, Correll CU, Curtis J, Gaughran F, Heald A, Hoare E, Jackson SE, Kisely S, Lovell K, Maj M, McGorry PD, Mihalopoulos C, Myles H, O'Donoghue B, Pillinger T, Sarris J, Schuch FB, Shiers D, Smith L, Solmi M, Suetani S, Taylor J, Teasdale SB, Thornicroft G, Torous J, Usherwood T, Vancampfort D, Veronese N, Ward PB, Yung AR, Killackey E, Stubbs B (2019). The Lancet Psychiatry Commission: a blueprint for protecting physical health in people with mental illness. Lancet Psychiatry.

[3] Vancampfort D, Firth J, Schuch FB, Rosenbaum S, Mugisha J, Hallgren M, Probst M, Ward PB, Gaughran F, De Hert M, Carvalho AF, Stubbs B (2017). Sedentary behavior and physical activity levels in people with schizophrenia, bipolar disorder and major depressive disorder: a global systematic review and meta-analysis. World Psychiatry.

[4] Scheewe TW, Jörg F, Takken T, Deenik J, Vancampfort D, Backx FJ, Cahn W (2019). Low physical activity and cardiorespiratory fitness in people with schizophrenia: a comparison with matched healthy controls and associations with mental and physical health. Front Psychiatry.

[5] Firth J, Solmi M, Wootton RE, Vancampfort D, Schuch FB, Hoare E, Gilbody S, Torous J, Teasdale SB, Jackson SE, Smith L, Eaton M, Jacka FN, Veronese N, Marx W, Ashdown-Franks G, Siskind D, Sarris J, Rosenbaum S, Carvalho AF, Stubbs B (2020). A meta-review of "lifestyle psychiatry": the role of exercise, smoking, diet and sleep in the prevention and treatment of mental disorders. World Psychiatry.

[6] Deenik J, Kruisdijk F, Tenback D, Braakman-Jansen A, Taal E, Hopman-Rock M, Beekman A, Tak E, Hendriksen I, van Harten P (2017). Physical activity and quality of life in long-term hospitalized patients with severe mental illness: a cross-sectional study. BMC Psychiatry.

[7] Czosnek L, Lederman O, Cormie P, Zopf E, Stubbs B, Rosenbaum S (2019). Health benefits, safety and cost of physical activity interventions for mental health conditions: a meta-review to inform translation efforts. Ment Health Phys Act.

[8] Stubbs B, Vancampfort D, Hallgren M, Firth J, Veronese N, Solmi M, Brand S, Cordes J, Malchow B, Gerber M, Schmitt A, Correll CU, De Hert M, Gaughran F, Schneider F, Kinnafick F, Falkai P, Möller HJ, Kahl KG (2018). EPA guidance on physical activity as a treatment for severe mental illness: a meta-review of the evidence and Position Statement from the European Psychiatric Association (EPA), supported by the International Organization of Physical Therapists in Mental Health (IOPTMH). Eur Psychiatry.

[9] Ashdown-Franks G, Williams J, Vancampfort D, Firth J, Schuch F, Hubbard K, Craig T, Gaughran F, Stubbs B (2018). Is it possible for people with severe mental illness to sit less and move more? A systematic review of interventions to increase physical activity or reduce sedentary behaviour. Schizophr Res.

[10] Brug J, Oenema A, Ferreira I (2005). Theory, evidence and intervention mapping to improve behavior nutrition and physical activity interventions. Int J Behav Nutr Phys Act.

[11] McEwan D, Beauchamp MR, Kouvousis C, Ray CM, Wyrough A, Rhodes RE (2019). Examining the active ingredients of physical activity interventions underpinned by theory versus no stated theory: a meta-analysis. Health Psychol Rev.

[12] Clausen W, Watanabe-Galloway S, Bill Baerentzen M, Britigan DH (2016). Health literacy among people with serious mental illness. Community Ment Health J.

[13] Fioravanti M, Bianchi V, Cinti ME (2012). Cognitive deficits in schizophrenia: an updated metanalysis of the scientific evidence. BMC Psychiatry.

[14] Harvey PD, Rosenthal JB (2018). Cognitive and functional deficits in people with schizophrenia: evidence for accelerated or exaggerated aging?. Schizophr Res.

[15] Vancampfort D, De Hert M, Stubbs B, Ward PB, Rosenbaum S, Soundy A, Probst M (2015). Negative symptoms are associated with lower autonomous motivation towards physical activity in people with schizophrenia. Compr Psychiatry.

[16] Ward MC, White DT, Druss BG (2015). A meta-review of lifestyle interventions for cardiovascular risk factors in the general medical population: lessons for individuals with serious mental illness. J Clin Psychiatry.

[17] Aleman A, Hijman R, de Haan EH, Kahn RS (1999). Memory impairment in schizophrenia: a meta-analysis. Am J Psychiatry.

[18] Rhodes RE, de Bruijn GJ (2013). How big is the physical activity intention-behaviour gap? A meta-analysis using the action control framework. Br J Health Psychol.

[19] Tangney JP, Baumeister RF, Boone AL (2004). High self-control predicts good adjustment, less pathology, better grades, and interpersonal success. J Pers.

[20] Friese M, Hofmann W, Wiers RW (2011). On taming horses and strengthening riders: recent developments in research on interventions to improve self-control in health behaviors. Self Identity.

[21] Hauser MD (2019). Patience! How to assess and strengthen self-control. Front Educ.

[22] Baumeister RF, Vohs KD, Tice DM (2007). The strength model of self-control. Curr Dir Psychol Sci.

[23] Finkel EJ, DeWall CN, Slotter EB, Oaten M, Foshee VA (2009). Self-regulatory failure and intimate partner violence perpetration. J Pers Soc Psychol.

[24] Beames JR, Schofield TP, Denson TF, de Ridder D, Adriaanse M, Fujita K (2018). A meta-analysis of improving self-control with practice. The Routledge International Handbook of Self-Control in Health and Well-Being.

[25] Friese M, Frankenbach J, Job V, Loschelder DD (2017). Does self-control training improve self-control? A meta-analysis. Perspect Psychol Sci.

[26] Hagger MS, Wood C, Stiff C, Chatzisarantis NL (2010). Ego depletion and the strength model of self-control: a meta-analysis. Psychol Bull.

[27] van Gemert-Pijnen LJ, Kip H, Kelders SM, Sanderman R, Kip H, Kelders SM, Sanderman R, van Gemert-Pijnen L (2018). Introducing eHealth. eHealth Research, Theory and Development: A Multidisciplinary Approach.

[28] Deenik J, Tenback DE, Tak EC, Blanson Henkemans OA, Rosenbaum S, Hendriksen IJ, van Harten PN (2019). Implementation barriers and facilitators of an integrated multidisciplinary lifestyle enhancing treatment for inpatients with severe mental illness: the MULTI study IV. BMC Health Serv Res.

[29] Drieschner KH, Boomsma A (2008). The Treatment Motivation Scales for forensic outpatient treatment (TMS-F): construction and psychometric evaluation. Assessment.

[30] Kelders SM, Kok RN, Ossebaard HC, Van Gemert-Pijnen JE (2012). Persuasive system design does matter: a systematic review of adherence to web-based interventions. J Med Internet Res.

[31] Ludden GD, van Rompay TJ, Kelders SM, van Gemert-Pijnen JE (2015). How to increase reach and adherence of web-based interventions: a design research viewpoint. J Med Internet Res.

[32] Kip H, Da Silva MC, Bouman YH, van Gemert-Pijnen LJ, Kelders SM (2021). A self-control training app to increase self-control and reduce aggression - a full factorial design. Internet Interv.

[33] Rhodes RE, Boudreau P, Josefsson KW, Ivarsson A (2021). Mediators of physical activity behaviour change interventions among adults: a systematic review and meta-analysis. Health Psychol Rev.

[34] Coon JC, Rapp JT (2018). Application of multiple baseline designs in behavior analytic research: evidence for the influence of new guidelines. Behav Interv.

[35] Carr JE (2005). Recommendations for reporting multiple-baseline designs across participants. Behav. Intervent.

[36] Krasny-Pacini A, Evans J (2018). Single-case experimental designs to assess intervention effectiveness in rehabilitation: a practical guide. Ann Phys Rehabil Med.

[37] Tate RL, Perdices M, Rosenkoetter U, Shadish W, Vohra S, Barlow DH, Horner R, Kazdin A, Kratochwill T, McDonald S, Sampson M, Shamseer L, Togher L, Albin R, Backman C, Douglas J, Evans JJ, Gast D, Manolov R, Mitchell G, Nickels L, Nikles J, Ownsworth T, Rose M, Schmid CH, Wilson B (2016). The Single-Case Reporting guideline In BEhavioural interventions (SCRIBE) 2016 statement. Remedial Spec Educ.

[38] Smith JD (2012). Single-case experimental designs: a systematic review of published research and current standards. Psychol Methods.

[39] Tate RL, Perdices M, Rosenkoetter U, Shadish W, Vohra S, Barlow DH, Horner R, Kazdin A, Kratochwill T, McDonald S, Sampson M, Shamseer L, Togher L, Albin R, Backman C, Douglas J, Evans JJ, Gast D, Manolov R, Mitchell G, Nickels L, Nikles J, Ownsworth T, Rose M, Schmid CH, Wilson B (2016). The Single-Case Reporting guideline In BEhavioural interventions (SCRIBE) 2016 statement. Arch Sci Psychol.

[40] Sieverink F (2017). Opening the black box of eHealth: a mixed methods approach for the evaluation of personal health records. University of Twente.

[41] Sieverink F, Kelders SM, van Gemert-Pijnen JE (2017). Clarifying the concept of adherence to eHealth technology: systematic review on when usage becomes adherence. J Med Internet Res.

[42] Dallery J, Cassidy RN, Raiff BR (2013). Single-case experimental designs to evaluate novel technology-based health interventions. J Med Internet Res.

[43] Denson TF, Capper MM, Oaten M, Friese M, Schofield TP (2011). Self-control training decreases aggression in response to provocation in aggressive individuals. J Res Pers.

[44] Kratochwill TR, Hitchcock JH, Horner RH, Levin JR, Odom SL, Rindskopf DM, Shadish WR (2013). Single-case intervention research design standards. Remedial Spec Educ.

[45] Urbaniak GC, Plous S (2013). Research Randomizer.

[46] Kratochwill TR, Hitchcock J, Horner RH, Levin JR, Odom SL, Rindskopf DM, Shadish WR (2010). Single‐case design technical documentation. What Works Clearinghouse.

[47] Delespaul PH, de consensusgroep EPA (2013). Consensus over de definitie van mensen met een ernstige psychische aandoening (EPA) en hun aantal in Nederland. Tijdschr Psychiatr.

[48] Lacey SJ, Street TD (2017). Measuring healthy behaviours using the stages of change model: an investigation into the physical activity and nutrition behaviours of Australian miners. Biopsychosoc Med.

[49] Prochaska JO, Velicer WF (1997). The transtheoretical model of health behavior change. Am J Health Promot.

[50] Schouten SE (2022). Best-practices, lessons learned and recommendations for the participatory design of eMental health with people with a severe mental illness: a qualitative multiple method approach. University of Twente.

[51] Oinas-Kukkonen H, Harjumaa M (2009). Persuasive systems design: key issues, process model, and system features. Commun Assoc Inf Syst.

[52] van Gemert-Pijnen L, Kelders S, Kip H, Sanderman R (2018). eHealth Research, Theory and Development: A Multidisciplinary Approach.

[53] (2016). Google Fit. GGD AppStore.

[54] Rubanovich CK, Mohr DC, Schueller SM (2017). Health app use among individuals with symptoms of depression and anxiety: a survey study with thematic coding. JMIR Ment Health.

[55] Santos-Lozano A, Santín-Medeiros F, Cardon G, Torres-Luque G, Bailón R, Bergmeir C, Ruiz JR, Lucia A, Garatachea N (2013). Actigraph GT3X: validation and determination of physical activity intensity cut points. Int J Sports Med.

[56] Sasaki JE, John D, Freedson PS (2011). Validation and comparison of ActiGraph activity monitors. J Sci Med Sport.

[57] Ozemek C, Kirschner MM, Wilkerson BS, Byun W, Kaminsky LA (2014). Intermonitor reliability of the GT3X+ accelerometer at hip, wrist and ankle sites during activities of daily living. Physiol Meas.

[58] Kruisdijk F, Deenik J, Tenback D, Tak E, Beekman AJ, van Harten P, Hopman-Rock M, Hendriksen I (2017). Accelerometer-measured sedentary behaviour and physical activity of inpatients with severe mental illness. Psychiatry Res.

[59] Deenik J, Tenback DE, Tak EC, Rutters F, Hendriksen IJ, van Harten PN (2019). Changes in physical and psychiatric health after a multidisciplinary lifestyle enhancing treatment for inpatients with severe mental illness: the MULTI study I. Schizophr Res.

[60] Ethica Data.

[61] van Berkel N, Ferreira D, Kostakos V (2017). The experience sampling method on mobile devices. ACM Comput Surv.

[62] Chang YJ, Paruthi G, Newman MW (2015). A field study comparing approaches to collecting annotated activity data in real-world settings. Proceedings of the 2015 ACM International Joint Conference on Pervasive and Ubiquitous Computing.

[63] Baumeister RF, Wright BR, Carreon D (2019). Self-control “in the wild”: experience sampling study of trait and state self-regulation. Self Identity.

[64] Bagala S (2021). Control yourself! How are trait and state self-control related to prosociality in young adults?. University of Twente.

[65] Schankweiler DA (2022). Resist the temptation: an interview study to explore and validate measures of psychotherapy clients’ self-control. University of Twente.

[66] Duckworth AL, Kern ML (2011). A meta-analysis of the convergent validity of self-control measures. J Res Pers.

[67] Fung SF, Kong CY, Huang Q (2019). Evaluating the dimensionality and psychometric properties of the brief self-control scale amongst Chinese university students. Front Psychol.

[68] de Ridder DT, de Boer BJ, Lugtig P, Bakker AB, van Hooft EA (2011). Not doing bad things is not equivalent to doing the right thing: distinguishing between inhibitory and initiatory self-control. Pers Individ Dif.

[69] Tangney JP, Boone AL, Baumeister RF, Baumeister RF (2018). High self-control predicts good adjustment, less pathology, better grades, and interpersonal success. Self-Regulation and Self-Control: Selected Works of Roy F. Baumeister.

[70] de Ridder DT, Lensvelt-Mulders G, Finkenauer C, Stok FM, Baumeister RF (2012). Taking stock of self-control: a meta-analysis of how trait self-control relates to a wide range of behaviors. Pers Soc Psychol Rev.

[71] Eigsti IM, Zayas V, Mischel W, Shoda Y, Ayduk O, Dadlani MB, Davidson MC, Lawrence Aber J, Casey BJ (2006). Predicting cognitive control from preschool to late adolescence and young adulthood. Psychol Sci.

[72] Vannest KJ, Parker RI, Gonen O, Adiguzel T (2016). Single Case Research: web based calculators for SCR analysis. (Version 2.0). Texas A&M University.

[73] R Core Team (2021). R: a language and environment for statistical computing. R Foundation for Statistical Computing.

[74] Iglewicz B, Hoaglin DC (1993). How to Detect and Handle Outliers.

[75] Peng CY, Chen LT (2018). Handling missing data in single-case studies. J Mod Appl Stat Methods.

[76] Parker RI, Vannest KJ, Davis JL, Sauber SB (2011). Combining nonoverlap and trend for single-case research: Tau-U. Behav Ther.

[77] Wilbert J (2021). Analyzing Single-Case Data with R and Scan. GitHub.

[78] Huitema BE, Mckean JW (2000). Design specification issues in time-series intervention models. Educ Psychol Meas.

[79] Braun V, Clarke V (2006). Using thematic analysis in psychology. Qual Res Psychol.

[80] Junger M, van Kampen M (2010). Cognitive ability and self-control in relation to dietary habits, physical activity and bodyweight in adolescents. Int J Behav Nutr Phys Act.

[81] Pfeffer I, Strobach T (2017). Executive functions, trait self-control, and the intention-behavior gap in physical activity behavior. J Sport Exerc Psychol.

[82] Lawrence D, Kisely S (2010). Inequalities in healthcare provision for people with severe mental illness. J Psychopharmacol.

[83] Merter S, Hasırcı D (2018). A participatory product design process with children with autism spectrum disorder. CoDesign.

[84] Kip H, Kelders SM, Bouman YH, van Gemert-Pijnen LJ (2019). The importance of systematically reporting and reflecting on eHealth development: participatory development process of a virtual reality application for forensic mental health care. J Med Internet Res.

[85] Kip H, Bouman YH, Kelders SM, van Gemert-Pijnen LJ (2018). eHealth in treatment of offenders in forensic mental health: a review of the current state. Front Psychiatry.

[86] Kip H, Oberschmidt K, Bierbooms JJ (2021). eHealth technology in forensic mental healthcare: recommendations for achieving benefits and overcoming barriers. Int J Forensic Ment Health.

[87] Orlowski SK, Lawn S, Venning A, Winsall M, Jones GM, Wyld K, Damarell RA, Antezana G, Schrader G, Smith D, Collin P, Bidargaddi N (2015). Participatory research as one piece of the puzzle: a systematic review of consumer involvement in design of technology-based youth mental health and well-being interventions. JMIR Hum Factors.

[88] Killikelly C, He Z, Reeder C, Wykes T (2017). Improving adherence to web-based and mobile technologies for people with psychosis: systematic review of new potential predictors of adherence. JMIR Mhealth Uhealth.

[89] Zhang MW, Ying J (2019). Incorporating participatory action research in attention bias modification interventions for addictive disorders: perspectives. Int J Environ Res Public Health.

[90] Scheepers F (2021). Mensen zijn ingewikkeld: een pleidooi voor acceptatie van de werkelijkheid en het loslaten van modeldenken.

[91] Firth J, Stubbs B, Vancampfort D, Schuch FB, Rosenbaum S, Ward PB, Firth JA, Sarris J, Yung AR (2018). The validity and value of self-reported physical activity and accelerometry in people with schizophrenia: a population-scale study of the UK biobank. Schizophr Bull.

[92] Soundy A, Roskell C, Stubbs B, Vancampfort D (2014). Selection, use and psychometric properties of physical activity measures to assess individuals with severe mental illness: a narrative synthesis. Arch Psychiatr Nurs.

[93] Stubbs B, Williams J, Gaughran F, Craig T (2016). How sedentary are people with psychosis? A systematic review and meta-analysis. Schizophr Res.

[94] Kwasnicka D, Inauen J, Nieuwenboom W, Nurmi J, Schneider A, Short CE, Dekkers T, Williams AJ, Bierbauer W, Haukkala A, Picariello F, Naughton F (2019). Challenges and solutions for N-of-1 design studies in health psychology. Health Psychol Rev.

[95] Julius RJ, Novitsky Jr MA, Dubin WR (2009). Medication adherence: a review of the literature and implications for clinical practice. J Psychiatr Pract.

[96] Liddelow C, Mullan B, Boyes M (2021). Understanding the predictors of medication adherence: applying temporal self-regulation theory. Psychol Health.

[97] Billen E, Garofalo C, Bogaerts S (2022). Self-regulation all bass-ackwards: similarities and differences in component structure in community and forensic psychiatric populations. Psychol Assess.

[98] Schulze LN, Stentzel U, Leipert J, Schulte J, Langosch J, Freyberger HJ, Hoffmann W, Grabe HJ, van den Berg N (2019). Improving medication adherence with telemedicine for adults with severe mental illness. Psychiatr Serv.

[99] Burton A, Marougka S, Priebe S (2010). Do financial incentives increase treatment adherence in people with severe mental illness? A systematic review. Epidemiol Psichiatr Soc.

[100] Petry NM, Rash CJ, Byrne S, Ashraf S, White WB (2012). Financial reinforcers for improving medication adherence: findings from a meta-analysis. Am J Med.

